# Plant-Growth Promoting *Bacillus oryzicola* YC7007 Modulates Stress-Response Gene Expression and Provides Protection From Salt Stress

**DOI:** 10.3389/fpls.2019.01646

**Published:** 2020-01-09

**Authors:** Dongwon Baek, Mohammad Rokibuzzaman, Ajmal Khan, Min Chul Kim, Hee Jin Park, Dae-jin Yun, Young Ryun Chung

**Affiliations:** ^1^Division of Applied Life Science (BK21plus program), Plant Molecular Biology and Biotechnology Research Center, Gyeongsang National University, Jinju, South Korea; ^2^Department of Biotechnology, Bacha Khan University, Charsadda, Pakistan; ^3^Department of Biomedical Science and Engineering, Konkuk University, Seoul, South Korea; ^4^Institute of Glocal Disease Control, Konkuk University, Seoul, South Korea

**Keywords:** *Bacillus oryzicola*, plant growth-promoting rhizobacteria, salt-stress tolerance, SOS pathway, malondialdehyde, sodium ion

## Abstract

High salt stress caused by ionic and osmotic stressors eventually results in the suppression of plant growth and a reduction in crop productivity. In our previous reports, we isolated the endophytic bacterium *Bacillus oryzicola* YC7007 from the rhizosphere of rice (*Oryza sativa* L.), which promoted plant growth and development and suppressed bacterial disease in rice by inducing systemic resistance and antibiotic production. In this study, *Arabidopsis thaliana* seedlings under salt stress that were bacterized with YC7007 displayed an increase in the number of lateral roots and greater fresh weight relative to that of the control seedlings. The chlorophyll content of the bacterized seedlings was increased when compared with that of untreated seedlings. The accumulation of salt-induced malondialdehyde and Na^+^ in seedlings was inhibited by their co-cultivation with YC7007. The expression of stress-related genes in the shoots and roots of seedlings was induced by YC7007 inoculation under salt stress conditions. Interestingly, YC7007-mediated salt tolerance requires SOS1, a plasma membrane-localized Na^+^/H^+^ antiporter, given that plant growth in *sos2-1* and *sos3-1* mutants was promoted under salt-stress conditions, whereas that of *sos1-1* mutants was not. In addition, inoculation with YC7007 in upland-crops, such as radish and cabbage, increased the number of lateral roots and the fresh weight of seedlings under salt-stress conditions. Our results suggest that *B. oryzicola* YC7007 enhanced plant tolerance to salt stress via the SOS1-dependent salt signaling pathway, resulting in the normal growth of salt-stressed plants.

## Introduction

Agricultural productivity cannot meet the requirements of an exploding world population despite distinct progress in farm management and agronomy; in addition, climate change affects agriculture and increases the risk of food insecurity (Nelson et al., 2014). In particular, soil salinity is one of the most representative environmental stressors and constitutes an important agriculture problem (Flowers, 2004; Van Oosten et al., 2017). Salinity has been shown to reduce the content of leaf chlorophyll, resulting in impaired photosynthesis under abiotic stress conditions (Chaves et al., 2009; Zhang et al., 2009a). Salt stress seriously damages photosynthesis as well as plant growth and development by resulting in water deficits, oxidant imbalances, cell-division impairment, membrane degeneration, ion injury, and osmotic stress (Abdel Latef et al., 2017; Abdel Latef et al., 2018; Abdel Latef et al., 2019a; Abdel Latef et al., 2019b). Plants have developed various mechanisms to avoid salt stress by employing compatible solutes which reduce sodium (Na^+^) uptake while regulating stomatal closure, shoot branching, and lateral root formation to conserve moisture (Acosta-Motos et al., 2017; Zhang et al., 2018). The expression of stress-response genes is regulated upon the initiation of unfavorable stress conditions (Shinozaki and Yamaguchi-Shinozaki, 2007; Hernández, 2019).

Plants heavily rely on two active defense mechanisms that involve ion transporters that either intensely exclude Na^+^ ions out of the cells or lock the ions into the vacuole (Zhu, 2002). The disintegration of ion homeostasis is the deleterious effect observed in plants that are exposed to salt stress (Munns and Tester, 2008). The salt overly sensitive (SOS) pathway is a key regulatory system that retains ion homeostasis during salt stress and *sos* mutants exhibit hypersensitivity to salt (Zhu, 2002). The SOS pathway involving the SOS1, SOS2, and SOS3 proteins, is essential for the maintenance of ion homeostasis (Zhu, 2002). Intercellular Na^+^ elevates intracellular Ca^2+^ levels, and these Ca^2+^ ions modulate sodium ion homeostasis together with the SOS proteins (Ji et al., 2013). Upon binding with Ca^2+^, SOS3 interacts with and activates SOS2 by releasing its self-inhibition. Then, the SOS3-SOS2 complex phosphorylates the plasma membrane protein SOS1, which effluxes Na^+^, reduces Na^+^ toxicity, and whose function is crucial to protect the root meristem (Ji et al., 2013). The high-affinity K^+^ transporter 1 (HKT1) functions as a major regulator that maintains the Na^+^ and K^+^ balance by regaining Na^+^ from the xylem in shoots (Rus et al., 2001).

Recently, it has been reported that plant tolerance can be enhanced by exogenous biostimulants, such as plant growth-promoting rhizobacteria (PGPR), to protect the plants against salt stress (Upadhyay and Singh, 2015; Habib et al., 2016; Li et al., 2017; Zou et al., 2018). Endophytic bacteria that are considered as PGPR have been applied to a broad range of agricultural crops to improve seed germination while increasing plant biomass and productivity (Gray and Smith, 2005; Paré et al., 2011; Bhattacharyya and Jha, 2012; Chung et al., 2015). The majority of PGPR include various strains of *Agrobacterium*, *Azospirillum*, *Bacillus*, *Pseudomonas*, and *Rhizobium* species (Hamdia et al., 2004; Bharti et al., 2013; Ahmad et al., 2014). These PGPR benefit plant growth through diverse mechanisms including nitrogen fixation; phosphate solubilization; and the production of 1-aminocyclopropane-1-carboxylate (ACC), deaminase, exopolysaccharide (EPS), and phytohormones, such as auxin, indole-3-acetic acid (IAA), abscisic acid (ABA), gibberellin (GA), and cytokinin (Cowan et al., 1999; Gyaneshwar et al., 2002; Mayak et al., 2004; Ryu et al., 2004; Figueiredo et al., 2008; Zhang et al., 2008; Yang et al., 2009).

*Bacillus subtilis* in *Arabidopsis thaliana* and *Rhizobium* and *Pseudomonas* in *Zea mays* increase plant tolerance to salt stress by regulating proline contents and ion homeostasis (Chen et al., 2007; Bano and Fatima, 2009). Lettuce leaves of *Lactuca sativa* inoculated with ACC-producing PGPR *Pseudomonas mendocina* exhibited enhanced plant tolerance to salt stress by increasing the uptake of nutrients and increasing the activity of antioxidant enzymes, such as peroxidase and catalase (Kohler et al., 2009). Brahmi (*Bacopa monnieri*) inoculated with EPS-producing PGPR *Bacillus pumilus* (STR2) and *Exiguobacterium oxidotolerans* (STR36) contained higher proline levels and lower lipid peroxidation levels in a saline environment (Bharti et al., 2013). The other multifarious bacteria, including *Pantoea*, *Paenibacillus*, *Burkholderia*, *Achromobacter*, *Microbacterium*, *Methylobacterium*, *Variovorax*, and *Enterobacter* species have also been reported as PGPR and mediate plant tolerance to various abiotic stressors, such as light, cold, heat, drought, salt, and oxygen (Choudhary, 2012; Kasotia and Choudhary, 2014).

Thus, the application of salt-tolerant PGPR to salt-hypersensitive crops, such as tomato, red pepper, maize, mung bean, and lettuce, might ensure plant growth and increase productivity during salt-stress conditions (Bano and Fatima, 2009; Tank and Saraf, 2010; Siddikee et al., 2011; Ahmad et al., 2012; Shukla et al., 2012; Ahmad et al., 2014). We previously reported the isolation and identification of an endophytic bacterium, *Bacillus oryzicola* YC7007, from the rice (*Oryza sativa* L.) rhizosphere (Chung et al., 2015). The YC7007 bacteria was found to promote plant growth and act as a biocontrol against fungal and bacterial rice pathogens through induced systemic resistance (ISR) and antibiotic production (Chung et al., 2015; Hossain et al., 2016). In this study, we investigated whether the PGPR *B. oryzicola* YC7007 ameliorates the adverse effects of high salt stress on the growth of *Arabidopsis thaliana* plants. The application of YC7007 may constitute an efficacious and sustainable approach for the agricultural management and protection of crops under salt-stress conditions.

## Materials and Methods

### Cultivation of the Endophytic Bacterium *Bacillus oryzicola* YC7007

The endophytic bacterium *B. oryzicola* YC7007 isolated from rice roots was used in the present study (Chung et al., 2015). The YC7007 strain was kept on tryptic soy agar (TSA; Difco Laboratories, Detroit, MI, USA) medium at 28°C for 24 h. The YC7007 cells were cultured in 1/10 X tryptic soy broth (TSB; Difco Laboratories, Detroit, MI, USA) at 28°C for 24 h and were adjusted to a 1 X 10^9^ colony forming unit (CFU)/ml in a 10 mM MgSO_4_ solution after centrifugation and washing in sterile distilled water for further experiments.

### Plant Materials and Growth Conditions

The ecotypes of *Arabidopsis thaliana* used in this study include Landsberg (Ler, *Landsberg erecta*), Wassilewskija (Ws-0), Colombia (Col-0), and a derivative of the Columbia C24 ecotype (TAIR, https://www.arabidopsis.org/). The *sos1-1*, *sos2-1*, and *sos3-1* mutants are described in Zhu et al. (1998). *Arabidopsis thaliana* seeds were surface-sterilized with 70% ethanol for 1 min followed by soaking for 3 min in 10% sodium hypochlorite and then washed with distilled water. The seeds were sown on 1/2 X Murashige and Skoog (MS) medium supplemented with 1.5% sucrose under short day conditions (8 h light / 16 h dark) at 23 °C. Surface-sterilized seeds from radish, *Raphanus sativus*, and cabbage, *Brassica campestris* ssp. *pekinensis*, were sown on 1/2 X MS medium that contained 1.5% sucrose under short day conditions at 28 °C.

### Treatment With *Bacillus Oryzicola* YC7007 and Salt

A total of 20 μl of the bacterial suspension of YC7007 that was adjusted to 10^9^ CFU/ml in 10 mM MgSO_4_ was used for the treatments. For horizontal growing, 5-day-old seedlings (10 seedlings per plate) were transferred onto one side of specialized plastic petri dishes containing a center partition (I plates; Fisher Scientific). Both sides of the petri dished contained 1/2 X MS with 1% sucrose and NaCl (0, 20, 40, 60, 80, or 100 mM). The treated plants were inoculated with either 50 µl of the bacterial suspension or 10 mM MgSO_4_ buffer at the center of the petri dish site opposite to the seedlings and grown for 14 days after incubation (DAI). For vertical growth, seedlings (10 seedlings per plate) were arranged in a line on one side of the petri dish. The other side of the petri dish was inoculated with a bacterial suspension at a distance of 5 cm from the seedling root tip on the opposite side of the petri dish. Plates were sealed with parafilm and placed in a growth chamber. Our experiments were performed independently at least 4 or 5 times with 6, 10, or 12 seedlings for each experiment.

The salt overly sensitive *sos1*, *sos2*, and *sos3* mutant seedlings were arranged in square plates (4 seedlings each) on one side of specialized square plastic petri dishes containing 1/2 X MS with 1% sucrose and either 30 or 50 mM NaCl and treated with 100 µl of the bacterial suspension at a distance of 5 cm from the primary root tip. The experiments were performed with six replicates with twelve seedlings per replicate.

### Analysis of Root Development

The root development parameters of horizontally grown seedlings were measured 14 days after inoculation (DAG). The growth of vertically grown seedlings was determined at 8 DAG. The fresh weight of the root and shoots was determined with an analytical balance. Primary root length was measured with a ruler and lateral root number was also determined immediately after harvesting. The dry weight of the seedlings was measured after being oven dried for 3 days at 80°C. The experiments were performed with five replicates with ten seedlings per replicate.

### Proton Concentration Measurements

The measurements of pH in the growth medium was determined by pH indicator (Zhang et al., 2009b). To quantify acidification of the plant growth medium, 4-day-old *Arabidopsis* (ecotype Col-0) seedlings were grown in 1/2 X MS medium with YC7007 or dH_2_O as a control for 5 days, then transferred to 1/2 X MS medium without agar. Before and after 24 h of roots or bacteria acidification, the pH value in growth medium was determined by a pH meter (Orion Star™ A211 pH Benchtop Meter; Thermo Scientific, Waltham, MA USA). The pH values without plant or YC7007 were used as blank controls for calculation of the proton concentration. Root proton release (unit: mol/g of fresh weight/h) was determined as (10^–final^ ^pH^ – 10^–initial^ ^pH^)*0.01/g of fresh weight of roots/24 h. The experiments were performed with three replicates of ten seedlings per replicate.

### Measurement of Chlorophyll Content

Leaf chlorophyll content was determined according to the methodology of Xie et al. (2009). 5 mg of tissue was pulverized with 1 ml 80% acetone and the absorbance of its supernatant was measured at 645 nm and 663 nm. Total chlorophyll content was calculated using the following formula:

$$Amount\;of\;chlorophyll\;mg\;g^{-1}=(7.15\times A_{663.2})+(18.71\times A_{646.8})/1,000/(fresh\;weight\;of\;leaves)$$

The experiments performed with three replicates with twenty seedlings per replicate.

### Measurement of Malondialdehyde

The oxidative stress biomarker malondialdehyde (MDA) was measured spectrophotometrically. Ground leaf tissue (0.1 mg) was mixed with 2 ml of 0.1% trichloroacetic acid (TCA) and centrifuged at 8,000 × *g* for 10 min. The supernatant was mixed with 20% TCA and 0.5% thiobarbituric acid (TBA) and incubated at 95°C for 30 min and then cooled immediately in an ice bath. After centrifugation, the absorbance of its supernatant was measured at 450 nm, 532 nm, and 600 nm to estimate the total leaf MDA content. The experiments were performed in triplicate with twenty seedlings per replicate.

### Visualization of Intracellular Na^+^ Ions and Ion Content Measurements

To visualize and measure the sodium ion content in the roots after NaCl treatment, we followed the methodology of Choi et al. (2011). Seven-day-old seedlings were treated with 200 mM NaCl and *B. oryzicola* YC7007 for 24 h. Then, the roots were stained with 20 μM CoroNa-Green in 0.02% pluoric acid for 3 h, and the fluorescence signals were observed using a GFP filter (excitation, 488 nm; emission, 510 nm) on a confocal laser-scanning microscope (Olympus FV1000; Olympus, Tokyo, Japan). Ten-day-old seedlings (n= 20) grown on 1/2 MS medium were transferred to the same medium supplemented with 200 mM NaCl and YC7007 for 6 h. The Na^+^ ion content was determined using inductively coupled plasma (ICP) spectrometry. The experiments were performed with three replicates of twenty seedlings per replicate.

### Analysis of Quantitative Real-Time PCR

Total RNA was isolated using a RNeasy Kit (Qiagen, Valencia, CA, USA) according to the instructions of the manufacturer and treated with DNase I (Qiagen, Valencia, CA, USA) to remove genomic DNA contamination. Total RNA (2 µg) was used for the first strand of cDNA synthesis using a cDNA synthesis kit (Invitrogen, Carlsbad, CA, USA) and subjected to qRT-PCR analysis. The primers used in the qRT-PCR analysis are described in **Supplementary Table 1**. QuantiMix SYBR (PhileKorea, Daejeon, Korea) was used for PCR reactions. The PCR conditions were 95°C for 10 min, 60 cycles of 95°C for 15 s, 60°C for 15 s, and 72°C for 15 s. The relative expression levels of all samples were automatically calculated and analyzed three times using CFX Manager Software (Bio-Rad, Hercules, CA, USA). The expression of *A. thaliana TUBULIN2* was used as the endogenous control. The qRT-PCR experiments were performed in triplicate. The specific primers of the stress-response genes used are listed in **Supplementary Table S1**.

### Statistical Analysis

The statistical analyses including Student’s t test were performed by Excel 2010 software. The qRT-PCR analyses were performed three-independent experiments, the average values of 2−ΔCT were used to determine the differences, and the data were expressed as means ± sd. A significant difference was considered at 0.01 < p-value ≤ 0.05 and p-value < 0.01.

## Results

### *Bacillus oryzicola* YC7007 Promoted the Growth of All *Arabidopsis* Ecotypes

**Figure 1:**
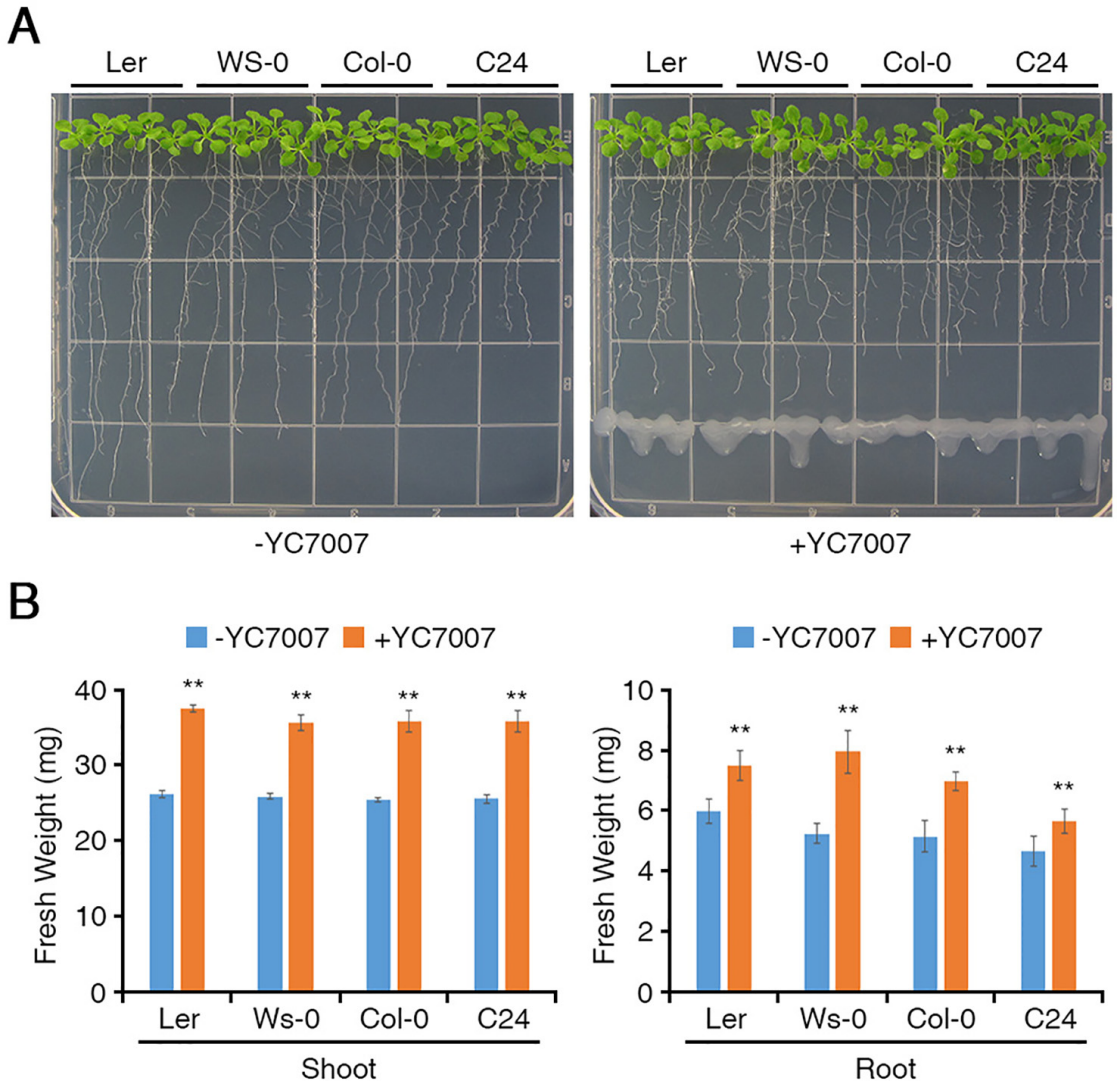
Comparison of plant growth among *Arabidopsis* Ler, Ws-0, Col-0, and C24 by an endophytic *Bacillus oryzicola* YC7007. Ler, Ws-0, Col-0 and C24 ecotype *Arabidopsis* plants were grown on 1/2 MS agar plate supplied with or without *B. oryzicola* YC7007. Suspension of *B. oryzicola* YC7007 in 10 mM MgSO_4_ was streak on the medium plate (+YC7007). The 10 mM MgSO4 solution was used as a control (Mock; -YC7007). **(A)** Photos were taken after 11 Days-After-Germination (DAG). **(B)** Fresh weight of shoot (left) and root (right), excising from the seedlings 11 DAG after *B. oryzicola* YC7007 treatment, were measured. The values indicated means ± SE of n = 3 replicates of 10 seedlings for each experiment. Asterisks represent significant differences from the untreated *B. oryzicola* YC7007 (-YC7007) (**, p-value < 0.01; Student’s t-test).

To investigate whether the YC7007 strain promotes plant growth, we used four ecotypes of *Arabidopsis*. At 11 DAG, the plant size of the seedlings co-cultivated with YC7007 was slightly greater than that of the non-treated control plants (**Figure 1**). The fresh weight of the seedling shoots and roots treated with YC7007 for 11 days was measured (**Figure 1B**). The shoot fresh weight of seedlings in the YC7007 treatment increased significantly by 37.7–43.4% when compared to that of the control seedlings (**Figure 1B**, left). The fresh weight of the roots in the YC7007 treatment also increased by 25.4% in Ler, 51.5% in Ws-0, 35.8% in Col-0, and 21% in C24 ecotypes compared to that of the control seedlings (**Figure 1B**, right).

The volatile organic compounds (VOCs) of PGPR induced acidification of the rhizosphere (Zhang et al., 2009b). To test whether VOCs of YC7007 was involved in rhizosphere acidification, we performed quantitative measurement of [H^+^] release in the growth medium before and after YC7007 treatment (**Table 1**). The values of pH in bacterial growth medium (TBS) and plant growth medium (MS) decreased by YC7007 (**Table 1**). 4-day-old *A. thaliana* ecotype Col-0 seedlings were initially grown in medium with exposure to H_2_O or YC7007 for 5 days. The [H^+^] release ability in root was enhanced approximately 5-fold with exposure to YC7007 than H_2_O (**Table 1**). These results suggested that YC7007 effectively increased rhizosphere acidification through by promoting root [H^+^] release.

**Table 1:** Acidification of growth medium.

Treatment	[H^+^] release (nmol/h)
Root acidification	
YC7007-treated plants	0.916 ± 0.067 g^-1^(FW)
H_2_O-treated plants	0.178 ± 0.022 g^-1^(FW)
Bacterial acidification	
TBS medium	0.58 ± 0.04 per treatment
MS medium	1.17 ± 0.03 per treatment

FW, fresh weight; YC7007, Bacillus oryzicola YC7007; TBS, 1/10 X Tryptic Soy Broth; MS, 1/2 X Murashige and Skoog.The experiments were performed with three replicates of ten seedlings per replicate.

### *Bacillus oryzicola* YC7007 Enhanced Plant Tolerance to Salt Stress

**Figure 2:**
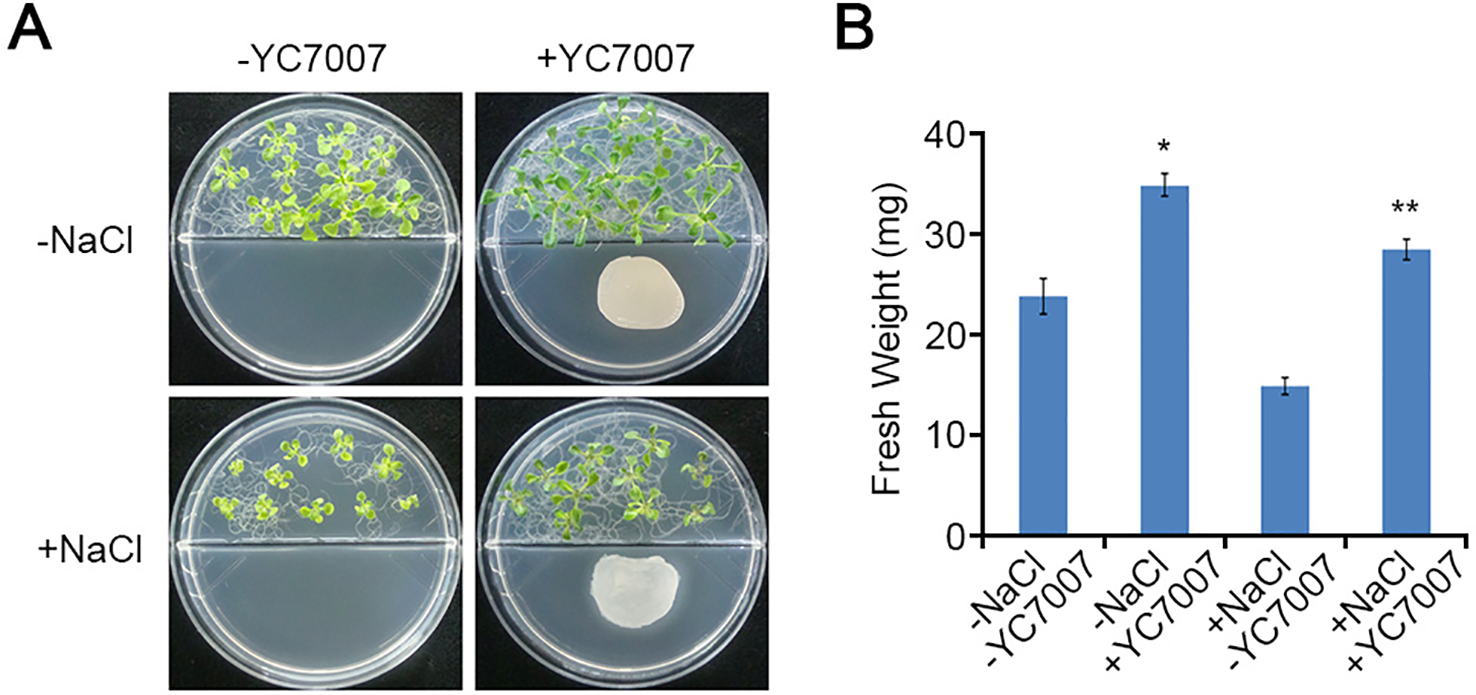
Effect of plant growth by *B. oryzicola* YC7007 during salt stress. Col-0 ecotype *Arabidopsis* plants were grown on 1/2 MS agar plate supplied with both 100 mM NaCl and *B. oryzicola* YC7007. Suspension of *B. oryzicola* YC7007 in 10 mM MgSO_4_ was streak on the medium plate with NaCl for co-cultivation. The 10 mM MgSO_4_ solution was used as a control (Mock; -YC7007). **(A)** Photos were taken after 14 Days-After-Inoculation (DAI). **(B)** Fresh weight of shoot, excising from the seedlings 14 DAI after both NaCl and *B. oryzicola* YC7007 treatment, were measured. The values indicated means ± SE of n = 5 replicates of 10 seedlings for each experiment. Asterisks represent significant differences from the untreated *B. oryzicola* YC7007 (-YC7007) (*, 0.01 < p-value ≤ 0.05; **, p-value < 0.01; Student’s t-test).

To test whether YC7007 has a protective effect in conditions of salt stress, we performed plant growth assays using *A. thaliana* ecotype Col-0 plants. At 14 DAI, the fresh weight of the shoots from the seedlings grown on salt MS media decreased approximately 37.8% compared to that of the untreated control shoots (**Figure 2**). Interestingly, the shoots from seedlings treated with YC7007 under salt stress conditions exhibited a 2-fold higher weight than untreated seedlings (**Figure 2**).

**Figure 3:**
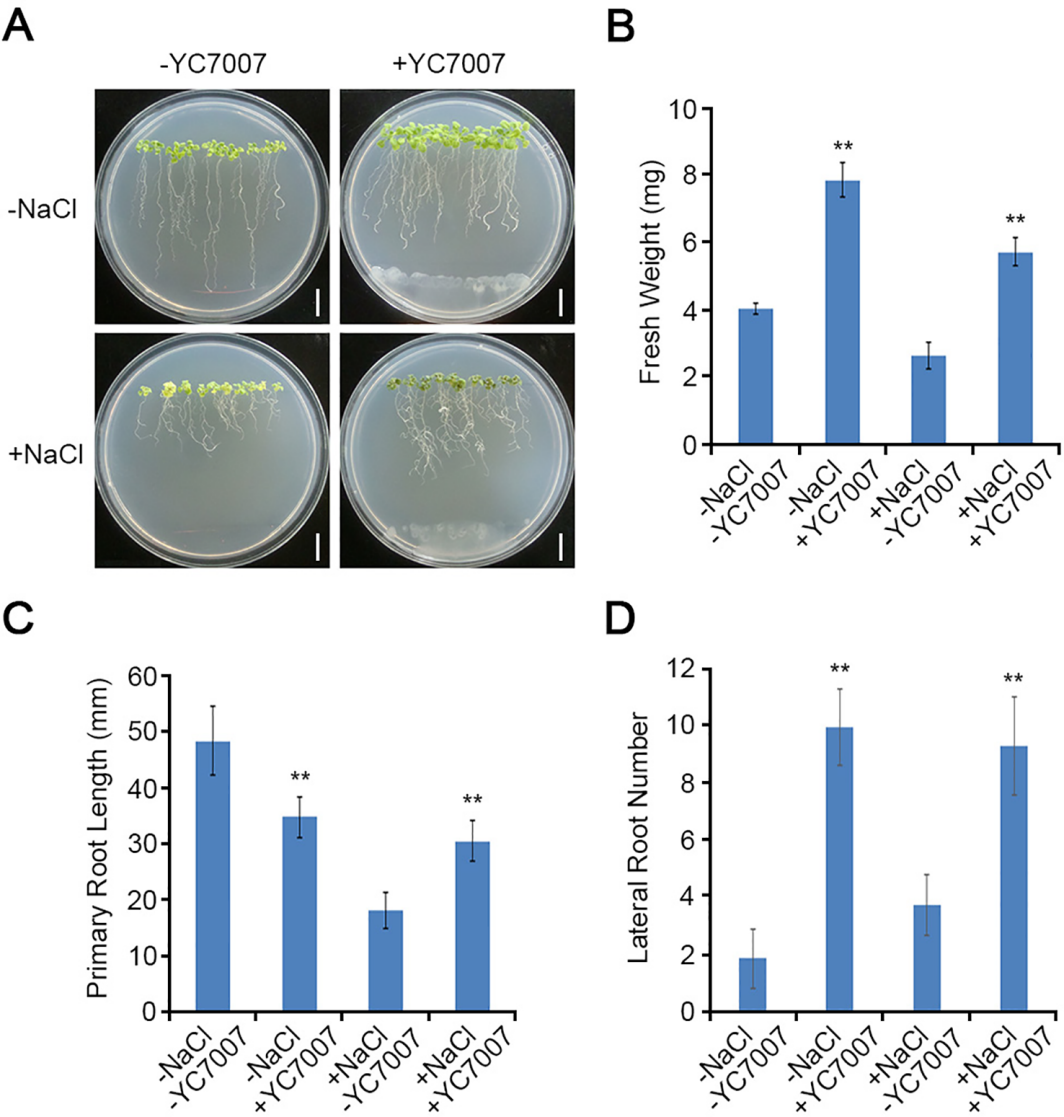
Effect of root development by *B. oryzicola* YC7007 during salt stress.Four-day-old seedlings of Col-0 ecotype *Arabidopsis* plants were transferred and vertically grown on 1/2 MS agar plate supplied with both 100 mM NaCl and *B. oryzicola* YC7007. **(A)** Roots of plants were photographed after 14 Days-After-Inoculation (DAI). Bar = 10 mm. **(B)** Fresh weight of roots, excising from the seedlings 14 DAI after both NaCl and *B. oryzicola* YC7007 treatment, were measured. **(C**, **D)** Changes in length of primary root **(C)** and number of lateral root **(D)** were analyzed in **(A)**. The values indicated means ± SE of n = 5 replicates of 10 seedlings for each experiment. Asterisks represent significant differences from the untreated *B. oryzicola* YC7007 (-YC7007) (**, p-value < 0.01; Student’s t-test).

To investigate the effects of YC7007 on plant root development under salt-stress conditions, we performed root growth assays. The weight of the roots was reduced by about 35.2% by the salt treatment when compared to that of the untreated control. The inoculation of YC7007 for 14 days increased the weight of salt-treated seedlings under salt stress 2.2-fold (**Figures 3A**, **B**). In addition, co-cultivation with YC7007 increased the length of the primary root and the number of lateral roots about 1.7- and 2.5-fold, respectively, under conditions of salt stress (**Figures 3C**, **D**). These results suggested that PGPR YC7007 enhanced plant tolerance to salt stress through the promotion of shoot and root development.

### *Bacillus oryzicola* YC7007 Increased the Content of Chlorophyll and MDA Under Salt Stress

**Figure 4:**
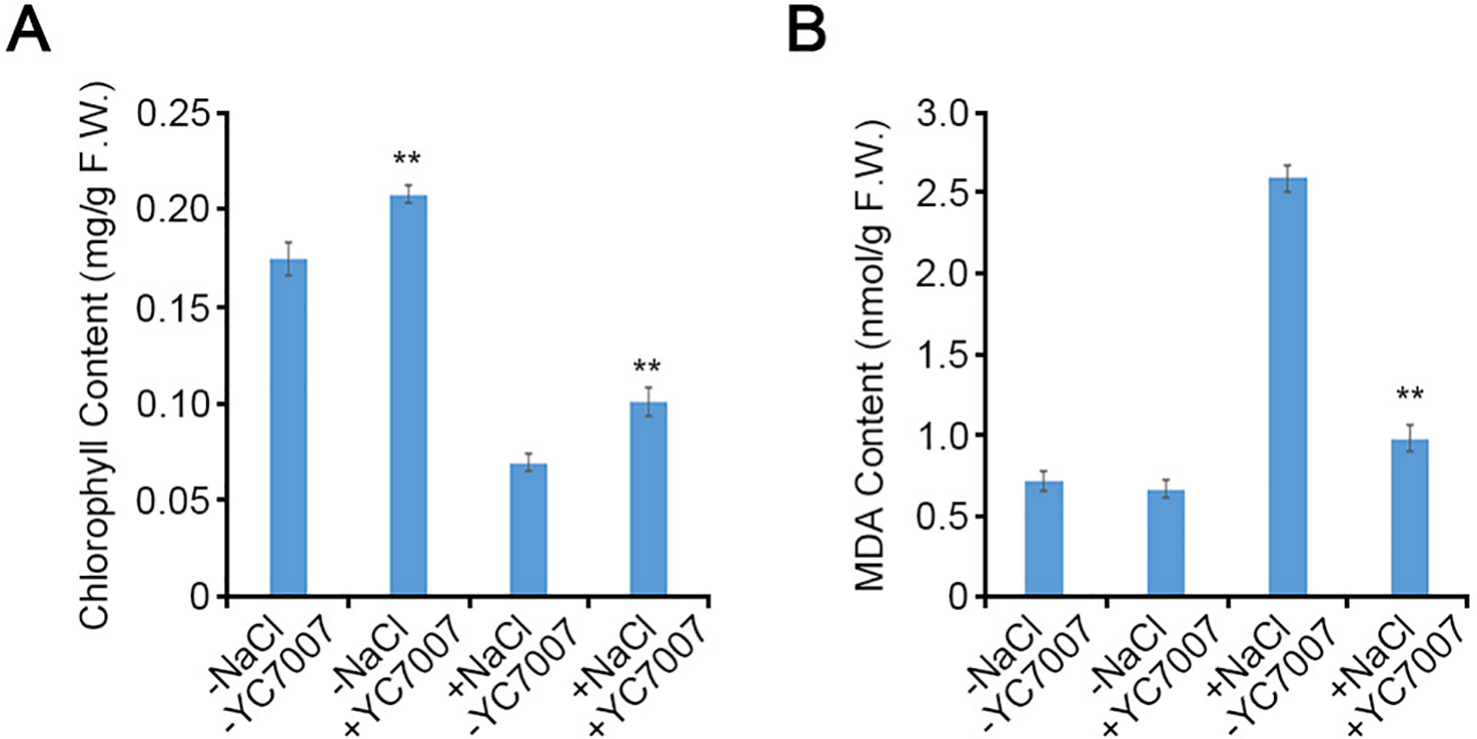
Quantitative comparison of chlorophyll and malondialdehyde (MDA) contents by *B. oryzicola* YC7007 during salt stress. Col-0 ecotype *Arabidopsis* plants were grown on 1/2 MS agar plate supplied with both 100 mM NaCl and *B. oryzicola* YC7007 after 14 Days-After-Germination (DAG). **(A)** 10 mg shoots from both NaCl- and *B. oryzicola* YC7007-treated seedlings were pulverized with acetone solution. The absorbance of samples was analyzed at wavelength of 645 nm and 663 nm. Total chlorophyll content was calculated using the following formula, as mg/g chlorophyll = (7.15 × A663 + 18.71 × A645) / 1000 / fresh weight of shoots. The values indicated means ± SE of n = 3 replicates of 10 seedlings for each experiment. **(B)** The MDA content in fresh weight of leaves was analyzed and compared between NaCl and *B. oryzicola* YC7007 treatment. The values indicated means ± SE of n = 3 replicates of 5 seedlings for each experiment. Asterisks represent significant differences from the untreated *B. oryzicola* YC7007 (-YC7007) (**, p-value < 0.01; Student’s t-test).

The *A. thaliana* Col-0 shoots from seeds cultivated on the 1/2 X MS containing 1.5% sucrose and streaked with YC7007 exhibited 20% more chlorophyll than untreated control plants at 14 DAG (**Figure 4A**). Under salt stress, the inoculation of YC7007 increased chlorophyll content by 45.4% compared to that of the control plants (**Figure 4A**). These results suggest that YC7007 promotes photosynthesis through the accumulation of chlorophyll under normal conditions and ensures its ability upon conditions of salt stress.

To investigate the change in MDA content due to treatment with YC7007 under salt-stress conditions, we measured a biomarker of lipid peroxidation (Kong et al., 2016). Even though there was no difference in MDA content under normal conditions, in the presence of salt, the MDA content of seedling shoots co-cultivated with YC7007 at 14 DAG decreased by about 62.3% compared to that of the untreated seedlings (**Figure 4B**). These results suggest that PGPR YC7007 improved plant tolerance to membrane oxidative damage under salt-stress conditions.

### *Bacillus oryzicola* YC7007 Maintained Na^+^ Homeostasis

**Figure 5:**
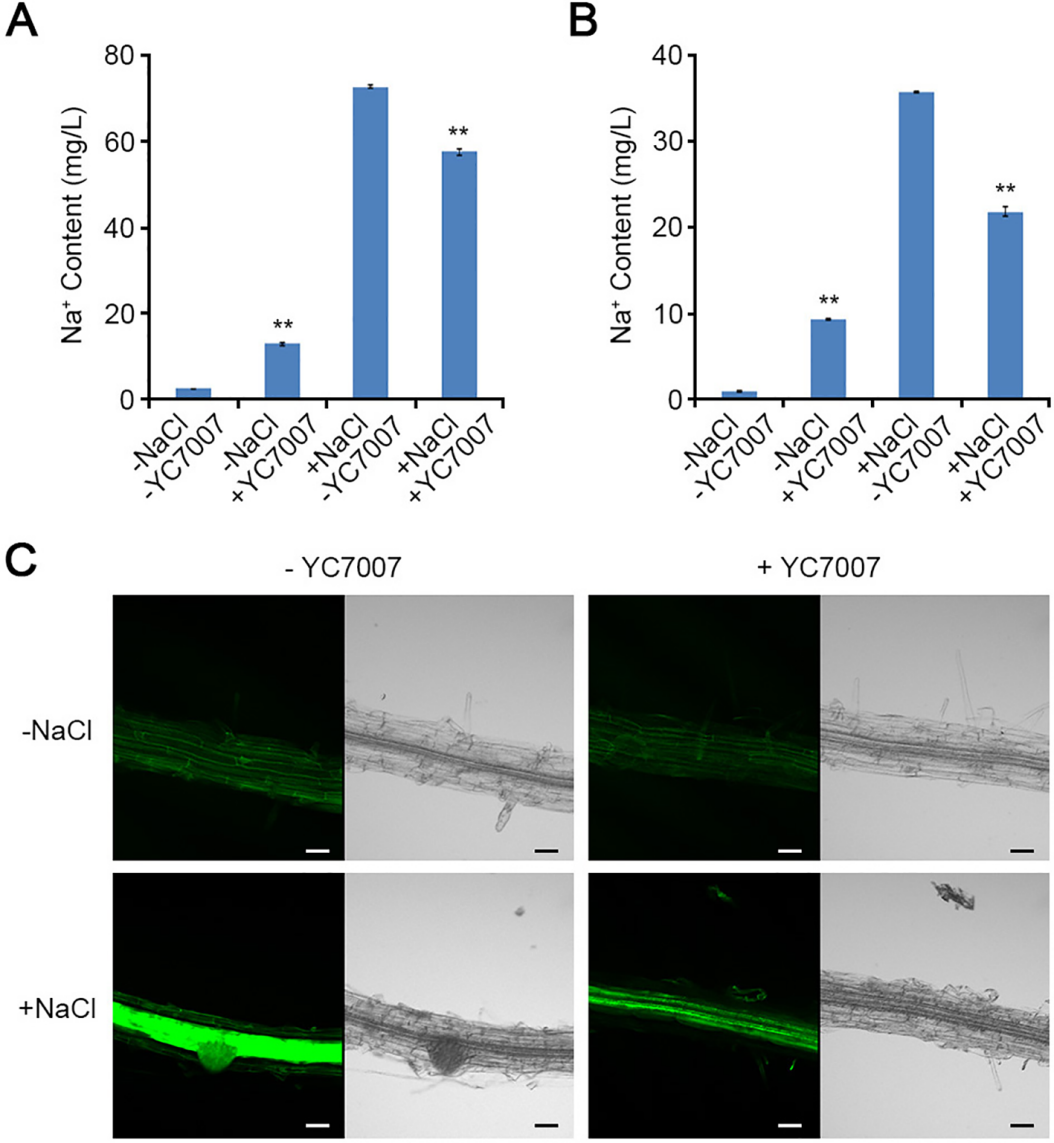
Effect of intracellular Na^+^ accumulation by *B. oryzicola* YC7007 during salt stress. **(A**, **B)** 10-day-old seedlings of Col-0 ecotype *Arabidopsis* plants were transferred 1/2 MS liquid medium supplied with both 200 mM NaCl and *B. oryzicola* YC7007. After 6 h, intracellular Na^+^ contents in shoots **(A)** and root **(B)** of Col-0 plants were measured by inductively coupled plasma (ICP) spectrometry. The values indicated means ± SE of n = 3 replicates of 10 seedlings for each experiment. Asterisks represent significant differences from the untreated *B. oryzicola* YC7007 (-YC7007) (**, p-value < 0.01; Student's t-test). **(C)** Visualization of intracellular Na^+^ by CoroNa-Green staining. 7-day-old seedlings of Col-0 ecotype *Arabidopsis* plants were transferred on filter papers supplied with both 200 mM NaCl and *B. oryzicola* YC7007. After 24 h, seedlings were stained with CoroNa-Green, and then visualized by confocal fluorescence microscopy. A representative image were photographed. Bar = 20 μm.

In the absence of salt, YC7007 treatment significantly increased the concentration of Na^+^ ion by about 5.4- and 10.4-fold in the shoots and roots, respectively (**Figures 5A**, **B**). However, in the presence of salt, YC7007 decreased Na^+^ ion content by about 20.9% and 38.9% in the shoots and roots, respectively (**Figures 5A**, **B**).

To visually confirm the effect of YC7007 in the control of Na^+^ ion content, a CoroNa-Green staining assay was performed. The dyes used indicators intracellular Na^+^ ions (Choi et al., 2011). The intensity of the fluorescent signal was reduced by YC7007 inoculation. However, the green fluorescence signals from the salt-treated roots without YC7007 were much stronger than those of YC7007-inoculated roots (**Figure 5C**). Taken together, these results indicate that YC7007 improved salt tolerance by controlling Na^+^ ion homeostasis *A. thaliana*.

### The Expression of Stress-Response Genes was Increased by *Bacillus oryzicola* YC7007 Under Salt-Stress Conditions

**Figure 6:**
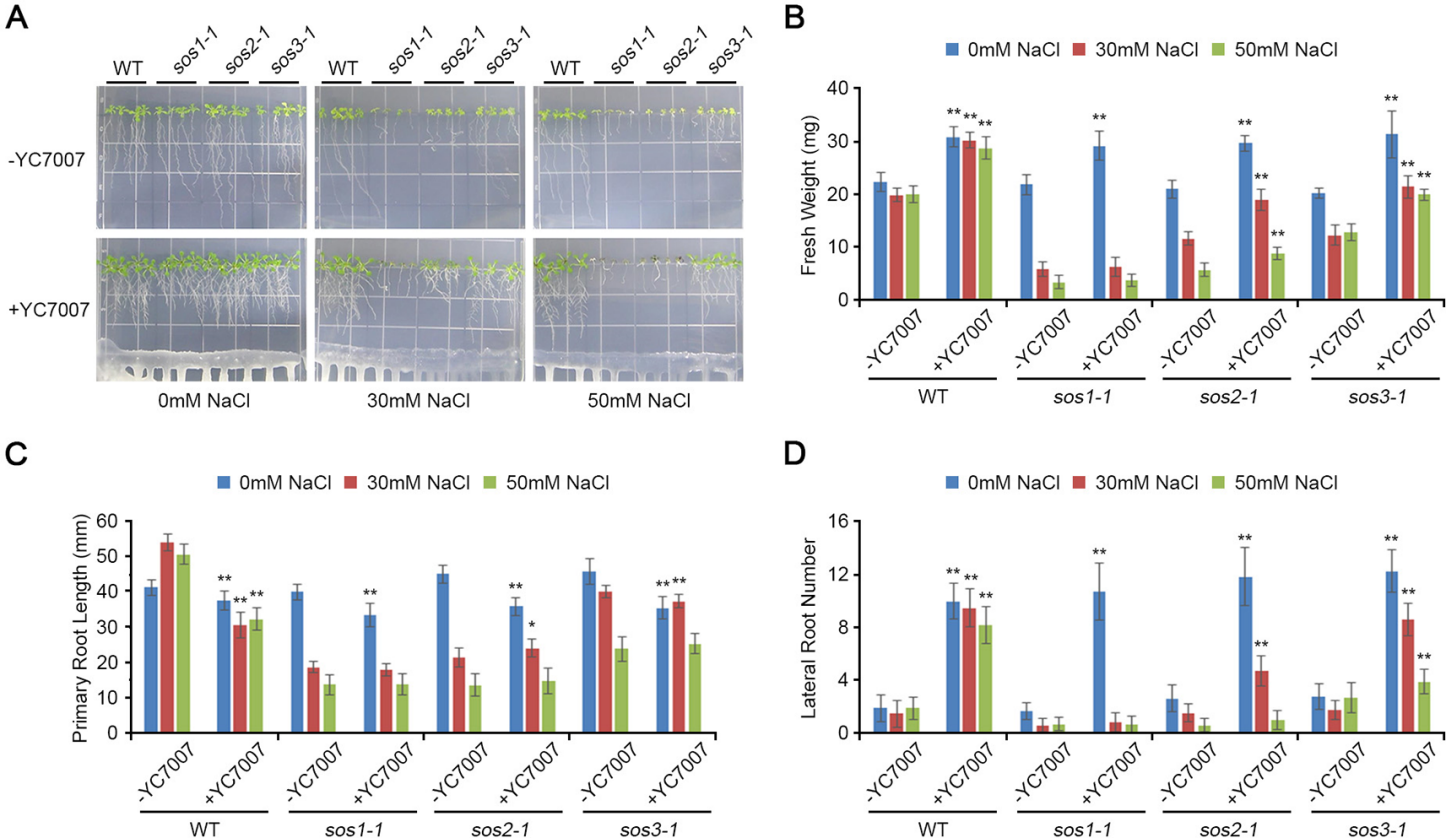
Comparison of plant growth among *Arabidopsis sos* mutants (*sos1-1*, *sos2-1*, and *sos3-1*) by *B. oryzicola* YC7007 during salt stress. Four-day-old seedlings of wild-type (WT), *sos1-1*, *sos2-1*, and *sos3-1* plants were transferred and vertically grown on 1/2 MS agar plate supplied with both different concentration (0 mM, 30 mM, and 50 mM) of NaCl and *B. oryzicola* YC7007. **(A)** Roots of plants were photographed after 14 Days-After-Inoculation (DAI). Bar = 10 mm. **(B)** Fresh weight of shoots, excising from the seedlings 14 DAI after both different concentration of NaCl and *B. oryzicola* YC7007 treatment, were measured. **(C**, **D)** Changes in length of primary root **(C)** and number of lateral root **(D)** were analyzed in **(A)**. The values indicated means ± SE of n = 4 replicates of 6 seedlings for each experiment. Asterisks represent significant differences from the untreated *B. oryzicola* YC7007 (-YC7007) (*, 0.01 < p-value ≤ 0.05; **, p-value < 0.01; Student’s t-test).

Under normal conditions, YC7007 enhanced the transcriptional expression of *RD29A*, *RD22*, *KIN1*, and *ERD1* in the roots (**Figure 6A**) and R*D29A* in the shoots (**Figure 6B**). Under salt-stress conditions, most stress-response genes (*RD29A, RD29B, RD20, RD22, KIN1*, and *ERD1*) were expressed strongly in the shoots and roots of seedlings co-cultivated with YC7007. These results indicated that YC7007 led to the up-regulation of stress-response genes under both natural and salt-stress conditions.

### *Bacillus oryzicola* YC7007 was Involved in the SOS-Dependent Pathway of the Induction of Salt Tolerance

**Figure 7:**
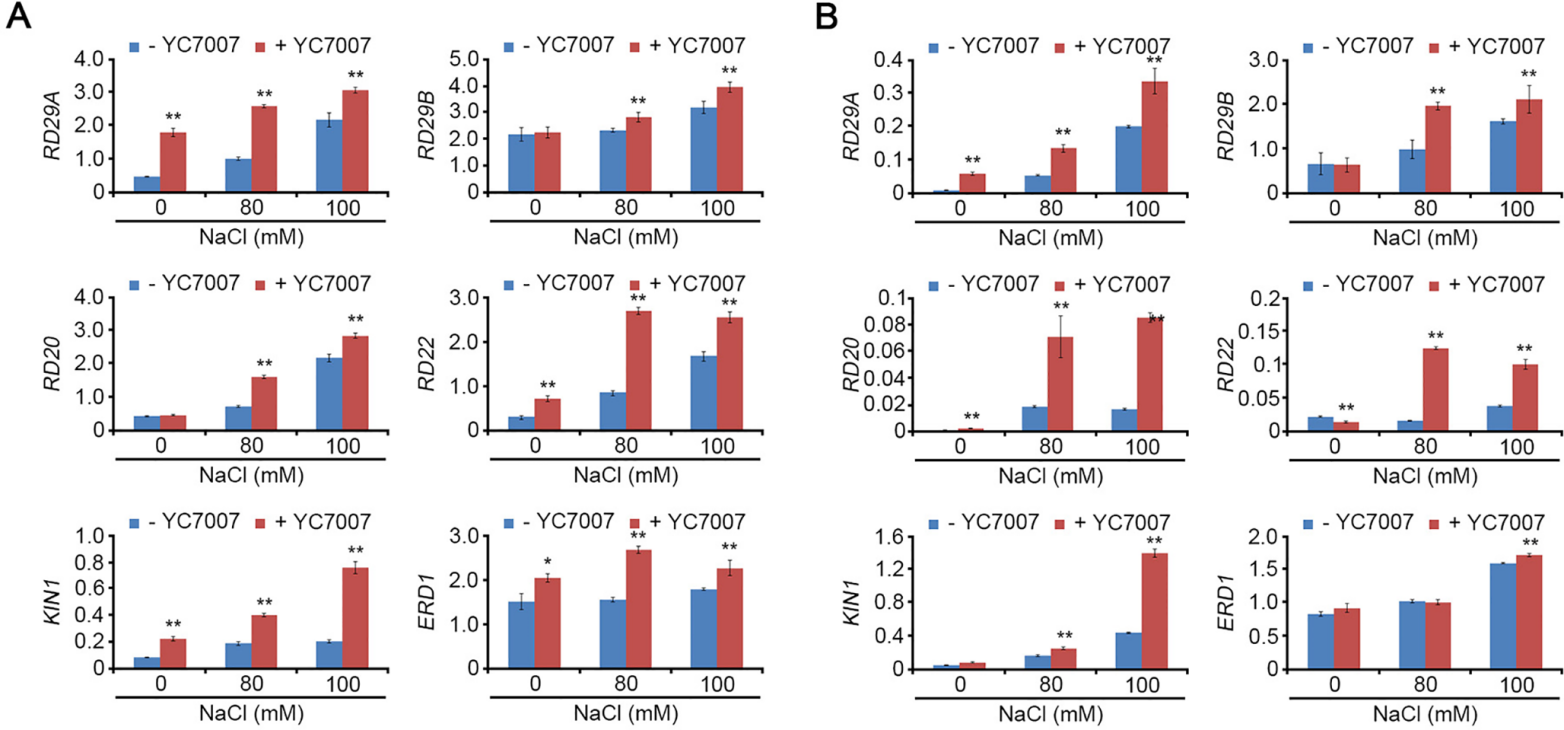
Effect of transcriptional expression of stress-responsive genes by *B. oryzicola* YC7007 during salt stress. 10-day-old seedlings of Col-0 ecotype *Arabidopsis* plants were transferred on 1/2 MS liquid medium supplied with both different concentration (0 mM, 80 mM, and 100 mM) of NaCl and *B. oryzicola* YC7007 for 6 h. Transcript levels of stress-responsive genes were measured by qRT-PCR in total RNA extracted from shoot **(A)** and root **(B)** of treated seedlings. Expression of *TUBULIN2* was used for normalization. Bars represent mean ± SD of three biological replicates with three technical replicates each. Asterisks represent significant differences from the untreated *B. oryzicola* YC7007 (-YC7007) (*, 0.01 < p-value ≤ 0.05; **, p-value < 0.01; Student’s t-test).

At 14 DAI, YC7007 increased the fresh weight and lateral root number of *sos* mutants but not primary root length under normal conditions (**Figure 7**). Upon conditions of salt stress, YC7007 alleviated the salt hypersensitivity of only *sos2-1* and *sos3-1* mutants, as reflected by an increase in their fresh weight and number of roots (**Figure 7**). Interestingly, the *sos1-1* mutants were not affected by co-cultivation with YC7007 (**Figure 7**). These data suggest that YC7007 might be involved in salt tolerance through SOS1-dependent signaling. The phenotype of *sos1-1* mutant to salt stress was more hypersensitive than that of *sos2-1* and *sos3-1* mutants (**Figure 6**) (Qiu et al., 2002; Qiu et al., 2004). The salt tolerance of *sos2-1* and *sos3-1* mutants by YC7007 were increased, suggesting that the functions of two genes in the same pathway to regulate by YC7007, unlike SOS1 function.

### *Bacillus oryzicola* YC7007 Enhanced Salt Tolerance in Vegetable Crops

**Figure 8:**
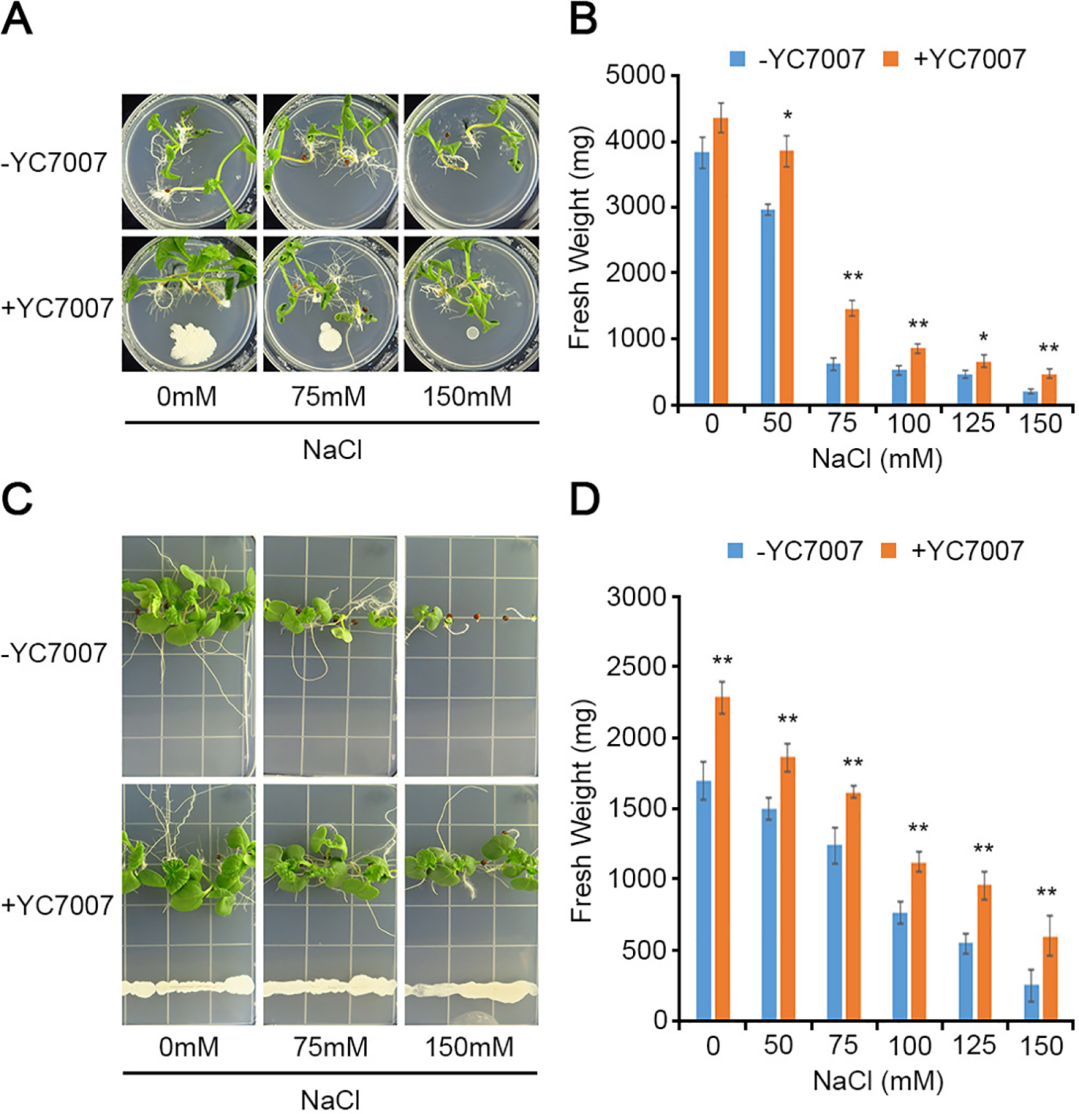
Comparison of plant growth among upland-crops, such as radish and cabbage, by *B. oryzicola* YC7007 during salt stress. The radish (*Raphanus sativus var*.; **A**, **B**) and cabbage (*Brassica campestris ssp. Pekinensis*; **C**, **D**) plants were grown on 1/2 MS agar plate supplied with both different concentration (0 mM, 75 mM, and 150 mM) of NaCl and *B. oryzicola* YC7007. **(A**, **C)** Radish **(A)** and cabbage **(C)** plants were photographed after 15 Days-After-Inoculation (DAI). **(B**, **D)** After both indicated different concentration of NaCl and *B. oryzicola* YC7007 treatment for 15 DAI, fresh weight of radish **(B)** and cabbage **(D)** seedlings were measured. The values indicated means ± SE of n = 3 replicates of 6 seedlings for each experiment. Asterisks represent significant differences from the untreated *B. oryzicola* YC7007 (-YC7007) (*, 0.01 < p-value ≤ 0.05; **, p-value < 0.01; Student’s t-test).

To understand the effect of YC7007 on vegetable crops in relation to salt tolerance, we evaluated radish and cabbage plants in our assays. At 15 DAG, the plant growth of the two crops was enhanced given the observed increase in fresh weight due to co-cultivation with YC7007 under both normal and salt-stress conditions (**Figure 8**). Notably, even under conditions of high salt stress (e.g., 150 mM NaCl), the inoculation of YC7007 increased the fresh weight of both radish and cabbage seedlings by about 2.34- and 2.37-fold, respectively, compared to that of the untreated control plants. These results indicate that YC7007 enhanced the plant growth, plant development, and salt tolerance of vegetables under salt-stress conditions.

## Discussion

The treatment of endophytic *B. oryzicola* YC7007 significantly promoted the growth and development of *A. thaliana* seedlings of all the tested ecotypes on 1/2 MS media given the observed increase in shoot and root weight. This bacterial strain has been previously found to promote rice growth while showing biological control activity against important seed-borne diseases (Chung et al., 2015). Some endophytic bacteria can be used as biocontrol agents for plant pathogens, insect, nematodes, and biostimulants to enhance plant growth and productivity (Santoyo et al., 2016). Furthermore, in this study we show that the *B. oryzicola* YC7007 strain enhanced the growth and the salt-stress tolerance of *A*. *thaliana* seedlings grown on 1/2 MS medium supplied with 100 mM NaCl given the increase in primary and lateral root development (**Figures 1**–**3**; Chung et al., 2015).

Various PGPR of different genera have been reported to induce salt-stress tolerance through the production of phytohormones, such as ACC deaminase, IAA, ABA, GA, and cytokinin (Nadeem et al., 2010; Numan et al., 2018). Plant growth-promoting rhizobacteria, such as *B. subtilis*, *Rhizobium*, and *Pseudomonas* species, have been found to increase plant tolerance to salt stress through the regulation of proline content and ion homeostasis in *A. thaliana* and *Z. mays* (Chen et al., 2007; Bano and Fatima, 2009). To reduce the impact of salt stress in plant, endophytic bacteria induce the accumulation of osmolytes and anti-oxidant compounds such as proline, glycine betaine, and anti-oxidant enzymes (Vaishnav et al., 2019). Rice inoculated with *P. pseudoalcaligenes* and *Bacillus pumilus* enhance salinity with higher level of glycine betaline (Jha et al., 2011). Volatile organic compounds (VOC) emitted from PGPR, which enter the atmosphere as vapors due to significantly high vapor pressure and low molecular weight (Vespermann et al., 2007; Dimkpa et al., 2009). VOCs from *B. subtilis* GB03 can stimulate many different hormonal signals which includes auxin, brassinosteroid cytokinin, GA and salicylic acid (SA) in *Arabidopsis* (Ryu et al., 2004). In addition, upon salt stress VOCs enhanced the plant tolerance of *Arabidopsis* to salt by regulating tissue-specific HKT1 expression, repressed in roots while upregulated in the shoots (Ryu et al., 2004; Zhang et al., 2008). Here, our study showed that the YC7007 strain enhanced plant growth and salinity tolerance (**Figure 2**). These salt stress responses alter the root architecture, especially the development of primary and lateral roots, by regulating cell division and differentiation (Vacheron et al., 2013; Etesami and Maheshwari, 2018; El-Esawi et al., 2018). The YC7007 strain has also been found to increase the fresh root weight, primary root length, and the number of lateral roots in *A. thaliana* plants under salt stress (**Figure 3**). Thus, YC7007 appears to influence the root system architecture via root developmental changes, which might enhance plant tolerance to salt stress. The changes in cellular root levels by YC7007 resulted in root formation, which was probably due to the production of VOCs emitted from YC7007 in response to salt stress as the bacterial cells did not touch the plant roots.

Plant growth-promoting rhizobacteria-mediated salt tolerance appears to include ABA- and SOS-mediated pathways and detoxification via the up-regulated expression of ABA-signaling cascade genes as well as the genes of antioxidant enzymes and osmolytes (Bharti et al., 2016). In our study, the salt sensitivity of *sos* mutants was examined under salt-stress conditions and co-cultivation with YC7007 to understand SOS-mediated salt tolerance. The YC7007 strain reduced salt accumulation both in the shoots and roots of *A*. *thaliana* seedlings (**Figure 5**). The salt hypersensitivity of the *sos1* mutants was not affected by YC7007, while *sos2* and *sos3* mutants exhibited increased salt-stress tolerance, suggesting that SOS1 is required for Na^+^ exclusion even in the presence of PGPR (**Figure 7**). The level of salt-induced MDA during salt stress was reduced by YC7007, which resulted oxidative damage (**Figure 4**). Moreover, genes involved in stress responses, such as *RD29A*, *RD29B*, *KIN1*, and *ERD1*, were expressed more in bacteria-treated plants under conditions of salt stress (**Figure 6**). The Na^+^/H^+^ transporter SOS1 was concerned in Na^+^ exclusion to salt stress response via alkalinization of intracellular pH and H^+^ efflux in *sos1* mutant (Guo et al., 2009). Our observations demonstrated that YC7007 was involved in rhizosphere acidification (**Table 1**). Taken together, YC7007 seems to induce salt tolerance via the SOS1-dependent signaling pathway under salt-stress conditions.

Each plant species has a different sensitivity to salt stress, although plant growth is eventually interrupted by high concentrations of salt in soil (Shannon and Grieve, 1999; Parida and Das, 2005). Many PGPR have been reported to enhance plant growth by enhancing stress tolerance in various crops, such as tomato, pepper, canola, bean, and lettuce during exposure to salt stress (Barassi et al., 2006). In particular, YC7007 enhanced the stress tolerance of radish and cabbage seedlings under conditions of salt stress, reflected in the observed increase in fresh weight under multiple salt-stress conditions (**Figure 8**).

We functionally characterized the role of *B. oryzicola* YC7007 in the SOS1-dependent signaling pathway involved in plant tolerance to salt stress. Our molecular, biological, and biochemical results suggest that *B. oryzicola* YC7007 regulates chlorophyll and MDA content while maintaining intracellular Na^+^ ion concentrations to enhance stress tolerance under conditions of salt stress. The physiological functions of the endophytic bacteria YC7007 are essential to understanding bacterial interactions that enhance plant growth and systemic tolerance to salt stress in plants as well as plant-bacteria communications. A clear understanding of the communication between PGPR and plants is more important than ever to ensure crop productivity and the development of economical crop management systems in agriculture.

## Data Availability Statement

All datasets generated for this study are included in the article/**Supplementary Material**.

## Author Contributions

YC and DB designed the experiments and wrote the manuscript. DB, MR, AK, and HP performed the experiments. MK and D-JY discussed the experimental results and commented on the manuscript.

## Funding

This study was supported by grant from the Next-Generation BioGreen21 Program (grant No. PJ 01104901 to YC and PJ01104902 to DB), Rural Development Administration Republic of Korea.

## Conflict of Interest

The authors declare that the research was conducted in the absence of any commercial or financial relationships that could be construed as a potential conflict of interest.
