# Supplementary material for: Plant-Growth Promoting Bacillus oryzicola YC7007 Modulates Stress-Response Gene Expression and Provides Protection From Salt Stress
Source: Front Plant Sci. 2020 Jan 9;10:1646. doi: 10.3389/fpls.2019.01646 (PMC6962239; doi:10.3389/fpls.2019.01646)
Supplement: Supplementary file 1 [file Table_1.docx]

***Supplementary Material***

**Plant-growth promoting Bacillus oryzicola YC7007 modulates stress-response gene expression and provides protection from salt stress**

Dongwon Baek^1^, Mohammad Rokibuzzaman^1^, Min Chul Kim^1^, Hee Jin Park^2,3^, Dae-jin Yun^2,3^, Young Ryun Chung^1*^

* Corresponding author:

Young Ryun Chung

Division of Applied Life Science (BK21plus program), Plant Molecular Biology and Biotechnology Research Center, Gyeongsang National University, Jinju 52828, Republic of Korea

E-Mail: [yrchung@gnu.ac.kr](mailto:yrchung@gnu.ac.kr)

**1. Supplementary Table**

**Supplemental Table S1.** Primer list for qRT-PCR in our study

| **Gene No.** | **Primer Name** | **Sequence (5’→3’)** |
| --- | --- | --- |
| AT5G62690 | TUBULIN2-qRT-F | TGGCATCAACTTTCATTGGA |
|  | TUBULIN2-qRT-R | ATGTTGCTCTCCGCTTCTGT |
| AT5G52310 | RD29A-qRT-F | CCTGAAGTGATCGATGCACCAG |
|  | RD29A-qRT-R | TGGTGTAATCGGAAGACACGAC |
| AT5G52300 | RD29B-qRT-F | GTGAAGATGACTATCTCGGTGG |
|  | RD29B-qRT-R | CACCACTGAGATAATCCGATCC |
| AT2G33380 | RD20-qRT-F | TTAGCTCCGGTCACCAGTCA |
|  | RD20-qRT-R | CATGTATGGTTTTGGTAATGTTTCC |
| AT5G25610 | RD22-qRT-F | ATGGCGATTCGGCTTCCTCTGATC |
|  | RD22-qRT-R | GACATTCATTTCTTTCCCGCGAAC |
| AT5G15960 | KIN1-qRT-F | CCAACAAGAATGCCTTCCAAGC |
|  | KIN1-qRT-R | GCTGCCGCATCCGATACACT |
| AT5G51070 | ERD1-qRT-F | CAGATGATGGAGATCTTGAA |
|  | ERD1-qRT-R | TGCGATCGATGTTTTGT |
